# *Ciliary Rootlet Coiled-Coil 2 (crocc2)* Is Associated with Evolutionary Divergence and Plasticity of Cichlid Jaw Shape

**DOI:** 10.1093/molbev/msab071

**Published:** 2021-03-15

**Authors:** Michelle C Gilbert, Emily Tetrault, Mary Packard, Dina Navon, R Craig Albertson

**Affiliations:** 1 Graduate Program in Organismic and Evolutionary Biology, University of Massachusetts, Amherst, MA, USA; 2 Graduate Program in Molecular and Cellular Biology, University of Massachusetts, Amherst, MA, USA; 3 Department of Biology, University of Massachusetts, Amherst, MA, USA

**Keywords:** craniofacial, cilia, mechanosensing, phenotypic plasticity, eco-devo

## Abstract

Cichlid fishes exhibit rapid, extensive, and replicative adaptive radiation in feeding morphology. Plasticity of the cichlid jaw has also been well documented, and this combination of iterative evolution and developmental plasticity has led to the proposition that the cichlid feeding apparatus represents a morphological “flexible stem.” Under this scenario, the fixation of environmentally sensitive genetic variation drives evolutionary divergence along a phenotypic axis established by the initial plastic response. Thus, if plasticity is predictable then so too should be the evolutionary response. We set out to explore these ideas at the molecular level by identifying genes that underlie both the evolution and plasticity of the cichlid jaw. As a first step, we fine-mapped an environment-specific quantitative trait loci for lower jaw shape in cichlids, and identified a nonsynonymous mutation in the *ciliary rootlet coiled-coil 2 (crocc2)*, which encodes a major structural component of the primary cilium. Given that primary cilia play key roles in skeletal mechanosensing, we reasoned that this gene may confer its effects by regulating the sensitivity of bone to respond to mechanical input. Using both cichlids and zebrafish, we confirmed this prediction through a series of experiments targeting multiple levels of biological organization. Taken together, our results implicate *crocc2* as a novel mediator of bone formation, plasticity, and evolution.

## Introduction

### Plasticity Is a Core Concept in the Extended Evolutionary Synthesis

The Modern Synthesis of the 1930s and 1940s united Darwin’s theory of natural selection with Mendelian genetics to explain the origin and maintenance of adaptive variation within populations, and has since been the prevailing paradigm in evolutionary biology (Mayr 1993). The Modern Synthesis set out a largely gene-centric view of adaptation wherein new variation arises in a population through genetic mutation, and natural selection leads to the differential survival of variants. In recent decades, however, it has become apparent that several elements are missing from the Modern Synthesis (Pigliucci 2007), including a consideration for previously unrecognized sources of variation, such as development (Waddington 1959; Jamniczky et al. 2010) and the environment (West-Eberhard 1989, 2003). In other words, the mechanisms for how phenotypic variation arises and is maintained within populations are not yet well understood (Hendrikse et al. 2007). These conceptual omissions have led to the idea that the field is in need of an Extended Evolutionary Synthesis (Mayr 1993; Pigliucci 2009).

Within the context of the Extended Evolutionary Synthesis, phenotypic plasticity has emerged as a core concept as it can have a potent effect on the degree and type of genetic variation that is exposed to natural selection (Mayr 1993; Pigliucci 2005, 2008; Laland et al. 2014). Operationally, plasticity is the capacity of a single genotype to produce two or more phenotypes in response to environmental stimuli (Bradshaw 1965), which may increase fitness in fluctuating environments (West-Eberhard 1989; Schlichting and Pigliucci 1998). Phenotypic plasticity also has the potential to influence several evolutionary phenomena, including the origins of novel traits (Moczek 2008), speciation (Price et al. 2003; West-Eberhard 2005; Pfennig et al. 2010), and adaptive radiations (West-Eberhard 2003; Wund et al. 2008). Although plasticity is often considered separate from (or even opposite to) genetics, it is important to note that the two are inextricably linked, and that plasticity manifests due to the sensitivity of (genetically encoded) molecules and/or pathways to environmental input (Pigliucci 2005). In other words, if phenotypic variance is due to the combined effects of genetics, the environment, and their interaction (i.e., P = G + E + GXE), then plasticity may be considered the interaction term (GxE). A genetic basis for plasticity is supported by its heritability (reviewed by, Scheiner 1993), but many questions remain, including what are the specific genetic components of plasticity and at what level (e.g., nucleotide, transcript, protein) do they confer environmental sensitivity (Pigliucci 2005; Gibert 2017). This uncertainty about the genetic nature of plasticity has hindered progress into understanding the mechanisms through which plasticity may evolve (Via et al. 1995). Thus, phenotypic plasticity is recognized as an important process in evolution, but we still lack an understanding of many fundamental aspects of its biology (Ehrenreich and Pfennig 2016; Schneider and Meyer 2017).

### The Cichlid Jaw as a Flexible Stem

Over the past two decades, we and others have worked to reveal the genetic bases for jaw shape differences among cichlid species (e.g., Albertson et al. 2003, 2005; Parnell et al. 2012; Powder et al. 2014; Hu and Albertson 2017; Irisarri et al. 2018). In addition, plasticity is well documented for the cichlid jaw. Specifically, when reared in the lab on diets that imposed distinct functional demands on the feeding apparatus, cichlids will develop distinct oral jaw morphologies (Bouton et al. 2002; van Snick Gray and Stauffer 2004). Notably, variation in cichlid feeding morphology induced by alternate feeding regimes can be strikingly similar to patterns of natural craniofacial variation among species (Parsons et al. 2014). Repeated lacustrine cichlid radiations are defined by a conserved primary axis of craniofacial variation that involves differences in head depth and jaw length/rotation, traits that are intimately associated with adaptations to alternate benthic and pelagic trophic habitats (Cooper et al. 2010), and it is this pattern of variation that is typically observed in studies of developmental plasticity of the cichlid jaw (Bouton et al. 2002; Parsons et al. 2014). Moreover, similar patterns of craniofacial plasticity have been observed in several other fish lineages when fed alternate benthic/pelagic diets (Parsons and Robinson 2006, 2007; Wund et al. 2008), which suggests a common mechanism may be at work.

The combination of morphological diversity and developmental plasticity has led to the assertion that the cichlid jaw represents a morphological “flexible stem” (West-Eberhard 2003). The flexible stem hypothesis of adaptive radiation postulates that patterns of developmental plasticity in an ancestral lineage will generate independently derived radiations along similar eco-morphological axes (West-Eberhard 1989, 2003). In other words, the nature of developmental plasticity in an ancestral population can influence the direction of adaptive radiations by determining what genetic variation is exposed to selection through its phenotypic expression. Under this hypothesis, repeated evolution along a conserved benthic-pelagic eco-morphological axis in cichlids is the result of sorting, and ultimately fixing, genetic variation that is exposed to selection via plasticity. If true, we would expect that the same loci that underlie evolutionary divergence in cichlid jaw shape will also regulate plasticity of the structure (Gibert 2017). Here, we test this prediction.

## Results and Discussion

### Genetic Variation in *crocc2* Is Associated with Functionally Salient Aspects of Cichlid Jaw Shape

**Fig. 1.**
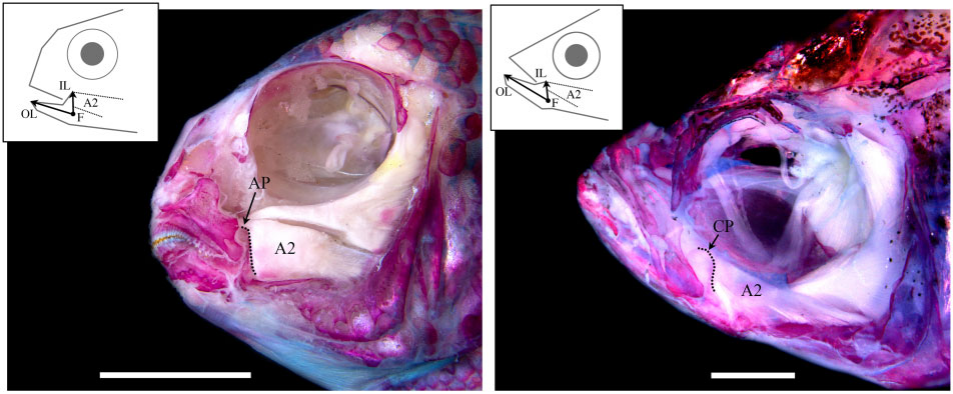
Functional anatomy of the cichlid and zebrafish head. A dissected and alizarin red stained head of a representative cichlid, *Tropheops* sp. “red cheek”, is depicted at left, and a zebrafish is shown at right. Craniofacial bones are red, and muscles are white. The lever mechanism that defines the mechanical advantage of jaw closing is illustrated for each species, whereby the jaw joint acts as the fulcrum (F), jaw length is the out-lever (OL), and a dorsally projecting bony process, on which the second subunit of the adductor mandibulae (A2) inserts, acts as the in-lever (IL). In cichlids, the in-lever is the ascending arm of the articular (AP), whereas in zebrafish it is the coronoid process (CP). Thus, in each species, this functional system is comprised of nonhomologous bony processes. Scale bar equals 1 cm in the cichlid image (left), and 1 mm in the zebrafish image (right).

We generated a hybrid mapping pedigree by crossing two cichlid species that differ in jaw shape as well as their ability to remodel their jaws under different environmental conditions (Parsons et al. 2014; Navon et al. 2020). The first species, *Labeotropheus fuelleborni* (LF hereafter), is an obligate algal scrapper, with a robust craniofacial skeleton and limited plasticity. The second, *Tropheops* sp. “red cheek” (TRC), is a more generalized benthic forager, with smaller jaws and greater plasticity. With this genetic cross, we mapped variation in feeding architecture under distinct, ecologically relevant feeding regimes, whereby families were split and progeny were made to feed with either “biting” or “sucking” modes of feeding, mimicking a major ecological axis of divergence (see Parsons et al. 2016 for details). Results from this study demonstrated that the craniofacial G–P map is strongly influenced by the environment, as most quantitative trait loci (QTL) were specific to one environment (Parsons et al. 2016; Zogbaum et al. 2021). Among the environmentally sensitive loci was a QTL for the mechanical advantage of jaw closing, which is defined as the height of the ascending arm of the articular bone (e.g., articular process, AP), relative to overall jaw length (fig. 1). In cichlids, this bony process is where a major muscle involved in jaw closing inserts (the second subunit of the adductor mandibulae, A2), establishing this structure as the in-lever for this functional system. A longer AP relative to jaw length, predicts greater mechanical advantage and a stronger bite. Lower jaw mechanical advantage tracks closely with feeding ecology in a range of vertebrate taxa (Westneat 2004; Manabu 2010; Roberts et al. 2011; Dumont et al. 2012; Casanovas-Vilar and van Dam 2013; Arbour and López-Fernández 2014), and is thought to drive evolutionary diversification (Dumont et al. 2014); however, its genetic basis is largely unknown (but see Albertson et al. 2005; Powder et al. 2014).

**Fig. 2.**
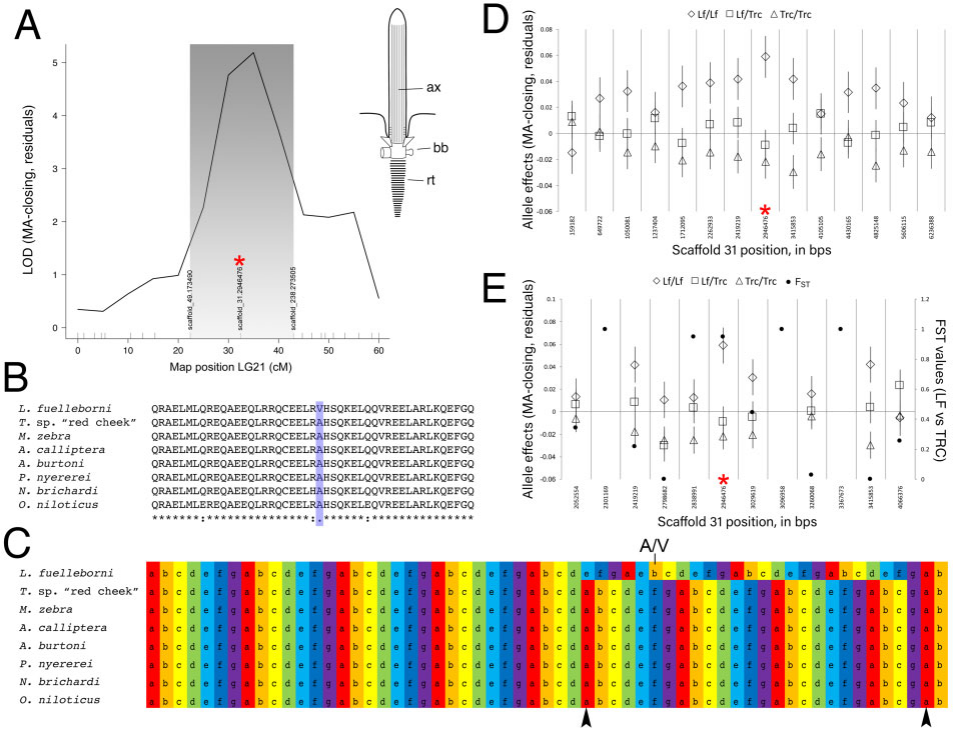
Mapping of lower jaw mechanical advantage in cichlids. The QTL for relative height of the articular process (i.e., mechanical advantage of jaw closing, “MA-closing”) maps to LG21 and peaks over a marker on physical scaffold number 31 (*A*). A schematic of a primary cilium is shown in (*A*) as well, where “ax” is the axoneme, “bb” is the basal body, and “rt” illustrates the striated rootlet. The SNP at the QTL peak (red asterisk) encodes a nonsynonymous (A/V) polymorphism within Crocc2, where the A allele is conserved across African cichlids (*B*), and is associated with two predicted interruptions (arrowheads, *C*) in the heptad repeat (i.e., denoted, and color-coded, a–g). The V allele in LF is predicted to result in contiguous heptad repeats in this region of the protein (*C*). With additional markers every ∼0.5 Mb, we queried the phenotype–genotype relationship along scaffold 31, and show that the peak association remains at ∼2.9 Mb (red asterisk, *D*). We sought to refine the interval even further using markers every ∼100-200 kb, between ∼2–4 Mb on scaffold 31, and find that the peak association holds at the *crocc2* SNP (red asterisk, *D*). Further, this marker is nearly alternatively fixed between -wild populations of LF and TRC (e.g., *F*_ST_ = 9.5).

In this genetic cross, relative AP height mapped to LG21 in the benthic/biting environment (but not the pelagic/suction feeding environment) (Parsons et al. 2016; fig. 2*A*). Fine mapping across the physical scaffold associated with the QTL interval showed that the peak genotype–phenotype association occurred at a SNP (i.e., G/A) within the *ciliary rootlet coiled-coil* 2 (*crocc2*) gene (fig. 2*B–D*). A genome scan for divergent loci between natural populations of the parental species used in this cross (i.e., LF and TRC) demonstrated that these species possess alternate *crocc2* alleles (i.e., *F*_ST_ = 0.95; fig. 2*D*; full data set published in Albertson et al. 2014). Notably, the SNP that underlied divergence within our mapping pedigree and between natural populations corresponded to a nonsynonymous change within *crocc2* (fig. 2*B*). This gene encodes an important structural component of the primary cilia, the ciliary rootlet. The alanine residue at position 963 appears to be conserved across African cichlids, but is a valine in LF, the obligate benthic forager with a long AP and low magnitudes of plasticity (fig. 2*B*). In addition, the A963V change is predicted to alter protein function based on a PolyPhen-2 (Adzhubei et al. 2013) protein prediction algorithm score of 0.904 (scores approaching 1.0 are considered functionally relevant), making this gene a robust candidate for regulating bone shape differences in cichlids.

*Crocc2* encodes a large protein composed almost entirely of coiled-coil domains (Yang et al. 2002). This structural motif forms alpha-helices through hydrophobic interactions, wherein the polypeptide chain coils in order to bury hydrophobic residues and expose polar side chains (reviewed by Woolfson [2005]). The pairing of coiled-coil proteins occurs through heptad repeats, usually denoted as *abcdefg*, where *a* and *d* represent the hydrophobic residues (fig. 2*E* and supplementary fig. S1*C*, Supplementary Material online), and interactions between opposing *a* and *d* residues represent the main hydrophobic seam in dimer formation (Woolfson 2005). In addition, residues that flank the hydrophobic seam in the alpha-helix, *e* and *g*, contribute to the specificity and stability between helices via ionic interactions (e.g., salt bridges). Coiled-coils are dynamic and flexible structural motifs, which participate in myriad biological functions.

In the cilium, Crocc2 monomers homodimerize to form filamentous rootlets, which originate from the basal body and extend proximally toward the cell nucleus (fig. 2*A*, inset). Rootlets are thought to provide structural support for cilia by integrating the cilium with actin filaments (Yang et al. 2005). Cells lacking rootlets are structurally unstable and degenerate over time (Yang et al. 2005; Mohan et al. 2013). Notably, the A963V change in African cichlids is predicted to affect this structural motif. Specifically, this change occurs in a stretch of residues where the heptad repeat is interrupted twice in African cichlids with the A alllele (black arrowheads, fig. 2*E*). The V allele in LF is predicted to re-establish the heptad repeat across this region (fig. 2*E*). Consistent with this, the stability of the dimerization between helices is predicted to be higher with the V allele (Tm = 95 °C), compared to the A allele (Tm = 85 °C) (bCIPA, Mason et al. 2006). Notably, dimerization between the two different alleles is predicted to be the least stable (Tm = 80 °C), which suggests that hybrids could be at a disadvantage if dimerization of this protein serves a core function. Collectively, these data suggest that this polymorphism may affect protein structure and cilia integrity/stability, with the V allele acting to increase stability.

When extending the Crocc2 sequence comparison across additional fish species several notable patterns emerged (supplementary fig. S1, Supplementary Material online). First, we found that all perciform species examined (*n* = 15) possessed either an A or V at this position, and further that all ray-finned fishes possessed a nonpolar, hydrophobic amino acid (supplementary fig. S1*B*, Supplementary Material online). In addition, the A/V polymorphism noted in noncichlid perciforms was associated with the same G/A nucleotide polymorphism. Thus, all species within this order seem to have one of two nucleotides at this position, leading to either an A or V, and correspondingly a stretch of Crocc2 characterized by interrupted or contiguous heptad repeats, respectively (supplementary fig. S1*C*, Supplementary Material online). The functional significance of this pattern with respect to bone/jaw shape remains unclear. On one hand, this region of the protein is characterized by increased variation in the continuity of the coiled-coil motif (relative to flanking regions), and so it may represent an area more permissive of variation, and therefore a potential target of selection. On the other hand, no obvious pattern emerges in terms jaw morphology when comparing species with continuous (e.g., LF, orangethroat darter, Antarctic dragonfish) versus interrupted (e.g., TRC, damselfishes, threespine stickleback) heptad repeats across this region. It is worth noting, however, that Crocc2 is a relatively large protein (>1,600aa in cichlids), and so it may be that the consequences of variation in amino acid sequence on bone biology has less to do with any one region of the protein, and more to do with the number and/or integrity of coiled-coil motifs across the entire protein, especially when making broad taxonomic comparisons. This could represent a fruitful line of future inquiry. Within African rift lake cichlids, however, where amino acid sequence homology is high (>95%), this particular mutation, and its predicted structural consequences, are more likely to have a direct effect on jaw/bone shape.

### Rates of Bone Matrix Deposition Are Canalized in the African Cichlid Species with the Divergent *crocc2* Allele

**Fig. 3.**
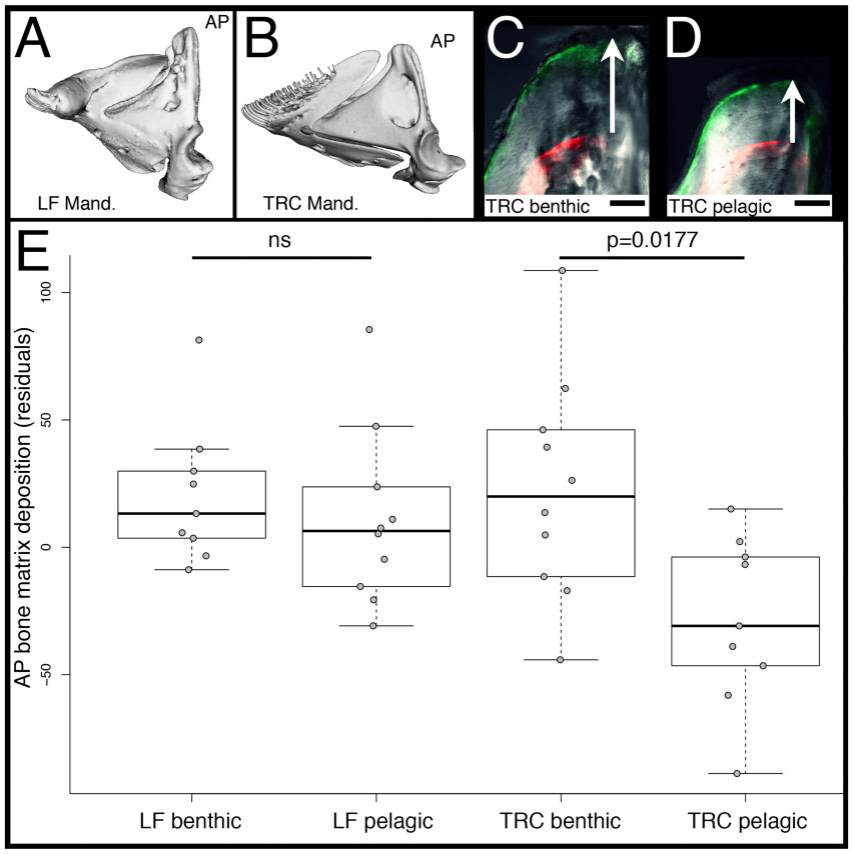
Rates of bone matrix deposition in cichlids. Mandibles of LF (*A*) and TRC (*B*) are shown, and the ascending arm of the articular bone (AP) is labeled. The tip of the AP in TRC reared in either a benthic/biting (*C*) and pelagic/sucking (*D*) environment is shown. Panels (*C* and *D*) are overlays of bright field, GFP, and RFP illumination. The RFP filter shows where alizarin red was incorporated into the bone. GFP is the calcein green label 5 weeks later. The distance between labels (white arrows) represents the amount of matrix deposited during that time. Scale bars equal 50 µm. Quantification of the rates of bone matrix deposition are shown in (*E*). Significance was determined via an ANOVA followed by a Tukey’s multiple comparison test.

In the context of plasticity, a mutation that influences the integrity of a structural protein could provide a mechanism through which genetic assimilation occurs. We know from previous work that the cichlid species with the divergent *crocc2* allele, LF, exhibits reduced craniofacial plasticity, relative to TRC, in response to alternate feeding regimes (Parsons et al. 2016; Navon et al. 2020). To determine the degree to which this finding holds specifically within the AP, we subjected LF and TRC to alternate feeding regimes, and then assessed rates of bone matrix deposition in the AP using two different fluorochromes injected at the beginning and end of the experiment (described in Navon et al. [2020]). We expected the generalized forager, TRC, to deposit more bone on the AP in the benthic/biting, compared with the pelagic/suction feeding, environment. Furthermore, we expected the obligate benthic foraging species, LF, to deposit relatively high rates of bone matrix deposition in both environments, consistent with the assimilation of a “biting” bone geometry. Our results support these predictions (fig. 3). We found a significant species-by-treatment effect in terms of matrix deposition (*F* = 4.108, *P* = 0.0137), with pairwise differences noted for TRC (TukeyHSD, *P* = 0.0177) but not LF (TukeyHSD, *P* = 0.9345) reared in different environments. These results show that bone formation is canalized in LF, resulting in consistently high levels of bone matrix deposition on the AP, and greater mechanical advantage of jaw closing.

Taken together, our results in cichlids suggest roles for *crocc2* in regulating species-specific bone geometry and plasticity, and that both phenotypes are related to differential mechanosensing. To test this hypothesis, we utilized the zebrafish system.

### *Crocc2* Is Required for the Maintenance of Primary Cilia

**Fig. 4.**
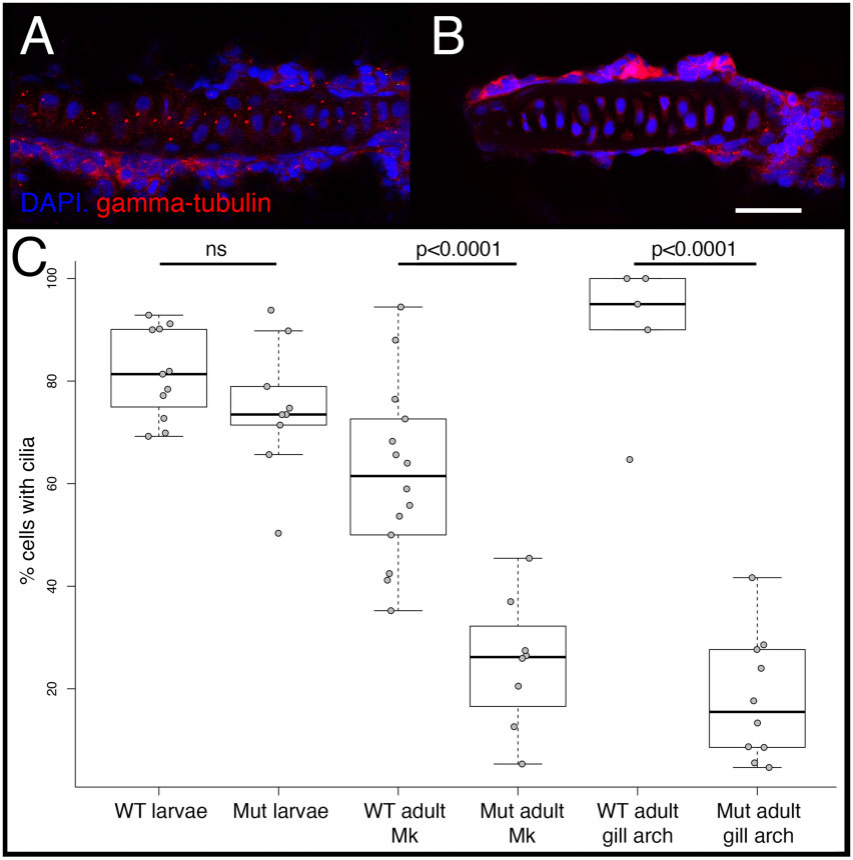
Cilia number in WT and mutant zebrafish. Cilia were visualized via immunohistochemistry using either anti-gamma-tubulin (shown), which labels the basal bodies, or anti-alpha acetylated-tubulin (not shown), which labels the axoneme, and imaged via confocal microscopy. Representative images are shown for the gill arch cartilage in WT (*A*) and full-sibling *crooc2* mutants (*B*). Scale bar equals 20 µm. Quantification of cilia number per cartilage, calculated as the percentage of nondividing cells containing cilia, is shown in (*C*). Significance was determined via an ANOVA followed by a Tukey’s multiple comparison test. In larval (4dpf) fish, each data point represents a count from a different cartilage across *n* = 3 WT and *n* = 3 *crocc2* mutant animals. In adults (>12 months), data points represent counts from different sections of Meckel’s cartilage (i.e., Mk), or from different gill arch cartilages. Sample sizes for adults are also *n* = 3 for each genotype.

Bone is a dynamic tissue that can sense and respond to its mechanical environment, and the primary cilia on bone cells are thought to play critical roles in mediating this process (Xiao et al. 2006; Papachroni et al. 2009; Nguyen and Jacobs 2013). Mice lacking functional cilia in bone precursor cells exhibit normal larval skeletal patterning, but impaired growth (Qiu et al. 2012), as well as a reduced ability to form bone in response to mechanical loading (Temiyasathit et al. 2012). Unlike the axoneme and basil body, roles for the ciliary rootlet in bone biology are unknown. The limited data on this structure suggest that rootlets are important for maintaining ciliary integrity over time (Yang et al. 2005). Consistently, we found that zebrafish *crocc2* mutants possessed primary cilia as larvae (e.g., 4 days), but exhibited a dramatic reduction in cilia number, compared to wild-type (WT) siblings, as adults (e.g., >12 months) (fig. 4). Based on these data as well as known roles for the primary cilia and bone mechanosensing, we predicted that *crocc2* mutants will exhibit bone phenotypes that include 1) dysmorphic skeletal architecture in areas of high mechanical stress, 2) degenerative bone homeostasis, and 3) a reduced ability to mechanosense.

### Jaw Defects in *crocc2* Mutants Localize to Regions of Adaptive Morphological Variation in the Cichlid Jaw

**Fig. 5.**
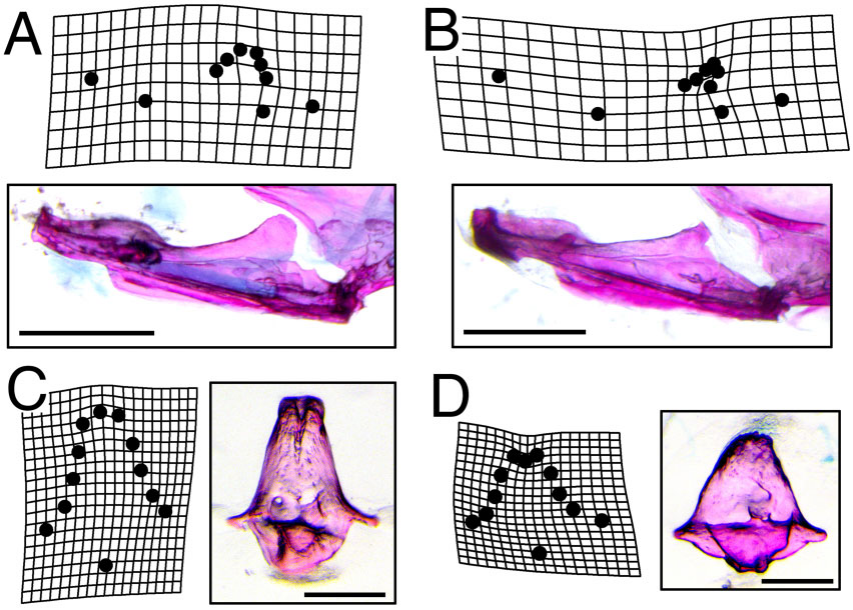
Dysmorphic bone geometry in *crocc2* mutants. A geometric morphometric shape analysis was performed on various element of the feeding apparatus in WT and *crocc2* mutant fish. Mutants exhibit distinct mandible shapes compared to WT siblings, with the most conspicuous differences occurring in the size and shape of the coronoid process (*B* vs. *A*). Scale bars in (*A*) and (*B*) equal 1 mm. Shape differences were also noted for the kinethmoid, with mutants exhibiting an overall shortening of the element in the dorsal–ventral dimension (*D* vs. *C*). Scale bars in (*C*) and (*D*) equal 200 µm. Deformation grids represent commonly seen phenotypes in the mandible, and exaggerated mean shapes in the kinethmoid. Procrustes ANOVA with post-hoc pairwise comparisons of group means (procD.lm, advanced.procD.lm), was significant for mandible mean shapes at *P* = 0.02, and for kinethmoid means at *P* = 0.12.

We found that homozygous recessive *crocc2* mutants were viable through adult stages, enabling the analysis of bone phenotypes throughout life history stages. Consistent with our prediction, patterning of the *crocc2* craniofacial skeleton appeared relatively normal, but shape was distinct, especially at adult stages. A geometric morphometric analysis of craniofacial shape revealed key differences in foraging related bones, specifically in regions with direct mechanical input (e.g., attachment points for tendons and ligaments) (fig. 5). For instance, variation that distinguished mutant and WT jaw shapes was largely limited to the size and shape of the coronoid process (CP, fig. 5*A* and *B*). In zebrafish, this structure represents the point of insertion for the A2 muscle (fig. 1), and is functionally analogous to the region of the cichlid jaw that maps to the *crocc2* locus. Thus, genetic/genomic mapping in cichlids and mutagenesis in zebrafish are consistent in implicating *crocc2* in the formation of nonhomologous but functionally equivalent structures of the jaw.

Shape defects were also noted in other bony elements. For example, the kinethmoid, which drives zebrafish jaw protrusion through a complex arrangement of ligamentous attachments, exhibited a unique shape in mutants (fig. 5*C* and *D*). Regions of this bone most affected in *crocc2* mutants include the rostral- and caudal-most surfaces, which serve as attachment sites for ligaments that connect the kinethmoid to the neurocranium and premaxilla, respectively (Staab and Hernandez 2010). In all, *crocc2* appears to be required to maintain bone integrity in zebrafish, especially in areas subjected to mechanical stress.

### *Crocc2* Is Required for Bone Homeostasis

We next examined bone growth and homeostasis in *crocc2* mutants at the transcript level. Specifically, we performed quantitative RT–PCR on freshly dissected craniofacial bones from mutant and WT animals at two different stages, young (3–5 months) and aging (10–15 months) adults. We used a panel of known and presumptive bone markers for this analysis (*n* = 10, supplementary table S1, Supplementary Material online), and reasoned that if 1) cilia are required for normal bone growth and homeostasis, and 2) *crocc2* mutants lose cilia over time, then we should observe a mis-regulation of bone marker genes in older, compared with younger, mutant animals.

When considering the expression of individual bone marker genes, we found evidence for mis-regulation (table 1 and supplementary fig. S2, Supplementary Material online). ANOVA models indicated significant effects of genotype on gene expression for three osteoblast markers (including *col10a1*), and all three Hedgehog (Hh) markers. Hh signaling was assessed given that members of this pathway localize to the primary cilia (Goetz et al. 2009), and that it plays important roles in bone development and homeostasis (reviewed by Long 2011; Alman 2015). Genotype was not significant for osteoclast markers, nor the mature chondroblast marker, *col2a1a.* Age also had a significant effect on the expression levels of 3/4 osteoblast genes, 2/3 Hh markers, as well as the osteoclast marker, *csf1ra.* Genotype-by-age was significant for the osteoblast markers, *runx2b* and *AP*, as well as for the Hh target gene, *ptch2.* The significant GxA effect for *runx2b* appeared to be driven by relatively higher expression in young mutant bones and lower expression in old mutant bones (supplementary fig. S2, Supplementary Material online). For *AP*, higher expression was documented in older mutant fish, compared with old WT and young mutants, whereas for the Hh markers, *ptch1* and *ptch2*, mutants exhibited relatively lower expression than WT at both stages (supplementary fig. S2, Supplementary Material).

**Table 1.** Expression Differences of Bone Marker Genes.

**Osteoblast markers:**	*Runx2b*		*Osx*		*AP*	
	F-value	P-value	F-value	P-value	F-value	P-value
Genotype	5.982	0.0163	2.796	0.0982	5.994	0.01621
Age	*31.757*	*1.78E-07*	3.23	0.0759	*11.466*	*0.00104*
GxA	*22.254*	*8.20E-06*	0.908	0.3435	*10.043*	*0.00206*

**Osteoclast markers:**	*Csf1ra*		*TRAP*			
	F-value	P-value	F-value	P-value		

Genotype	2.824	0.097185	1.567	0.214		
Age	*13.951*	*3.73E-04*	2.589	0.111		
GxA	1.557	0.216196	0.381	0.539		
						

**Chondroblast markers:**	Col10a1*		*Col2a1a*			
	F-value	P-value	F-value	P-value		

Genotype	3.986	0.048744	0.045	0.8317		
Age	*12.967*	*5.06E-04*	4.929	0.0291		
GxA	0.009	0.925719	0.217	0.6424		
						

**Hedgehog markers:**	*Ptch1*		*Ptch2*		*Gli*	
	F-value	P-value	F-value	P-value	F-value	P-value

Genotype	6.758	0.0111	*18.683*	*6.39E-05*	5.373	0.0233
Age	*6.351*	*1.37E-02*	*18.93*	*5.80E-05*	0.845	0.361
GxA	1.09	0.2996	4.324	0.0422	0.04	0.8415

Note.—Expression of genes involved in bone/cartilage development was assessed in WT and mutant animals at two life-history stages, young adult (3–5 months) and old adult (10–15 months). The ANOVA model was (expression ∼ genotype × age), and the effects of genotype, age, and their interaction are presented. Marker genes are organized by general function. *Col10a1* has an asterisk next to it, because it plays roles in both endochondral and dermal bone formation in fishes. Values with significance after Bonferroni-correction are italicized.

**Fig. 6.**
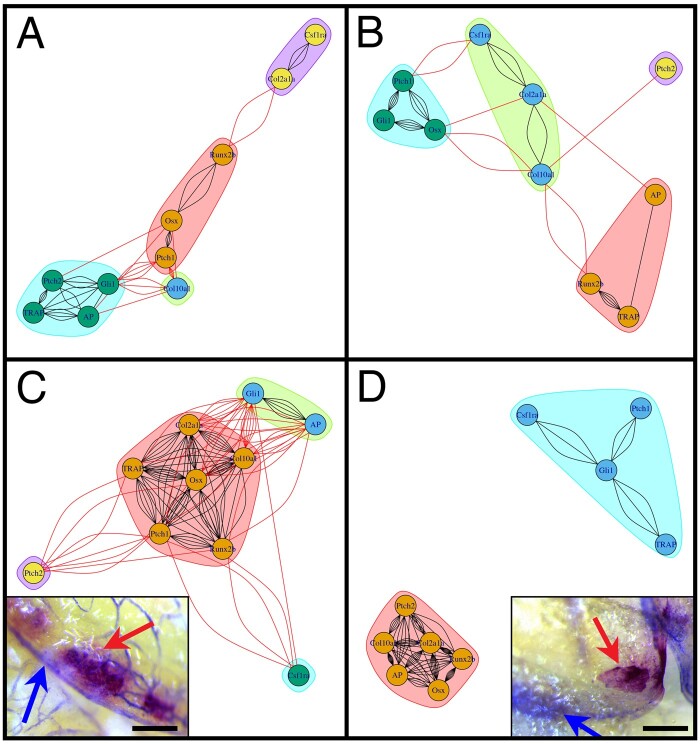
Mis-regulation of the bone marker gene expression in *crocc2* mutants. Network output of partial correlations (from table 2). Red lines represent correlations between genes in different modules, whereas black lines represent correlations within modules. Colors denote distinct modules in each analysis. Panel (*A*) illustrates the interaction between bone marker expression in WT animals at the young adult stage (3–5 months), whereas panel (*B*) shows data for comparably staged mutants. Note that, although there are a greater number of correlations in WT versus *crocc2* mutant animals, both networks are characterized by four interconnected modules. Covariation of gene expression in old adult (10–15 months) bone is shown for WT (*C*) and mutant (*D*) animals. WT zebrafish show a relatively high number of correlations both within and between modules, consistent with a tightly integrated gene network. Alternatively, mutants show a dissociated pattern characterized by two distinct modules, which is reflected in in vivo patterns of bone cell activity (insets, *C* and *D*). In WT bone (i.e., interopercle), TRAP and AP are generally in close approximation, whereas in mutants these factors are often expressed in distinct areas of the bone. Scale bars equal 200 µm.

As bone homeostasis requires the coordinated expression of multiple genes, we next sought to assess the degree to which these genes exhibited coordinated expression in mutant and WT animals. Specifically, we performed partial correlations analyses on expression data within mutant and WT animals at both life-history stages, and report correlation coefficients and *P* values for all pairwise comparisons with the effect of the other variables removed (table 2). Among young adults, differences between genotypes were modest, with mutants exhibiting 8/45 significant (*P* < 0.05) pairwise correlations, compared with 11/45 in similarly aged WT siblings (table 2). Further, of the 11 significant correlations in WTs, only three were shared with mutants. This pattern was reflected in a network analyses of expression data, where both WT and mutant animals were characterized by four modules of correlated gene expression; however, the composition of genes within each module was different, as was the overall strength of correlation in gene expression, which was higher in WT bone (i.e., a greater number of lines connecting traits, fig. 6*A* and *B*).

**Table 2.** Covariation in the Expression of Bone Marker Genes.

**Young WT Bone**										
	*runx2b*	*col10a1*	*AP*	*TRAP*	*osx*	*ptch1*	*col2a1a*	*csf1ra*	*gli1*	*ptch2*
*runx2b*		0.413	0.637	0.757	*0.022*	0.480	0.080	0.869	0.830	0.343
*col10a1*	0.200		0.119	0.630	0.114	*0.003*	0.654	0.558	*0.040*	0.124
*AP*	−0.116	0.370		*0.004*	0.098	0.509	0.200	0.814	0.115	*0.040*
*TRAP*	0.076	−0.118	*−0.622*		0.356	0.649	0.922	0.426	*0.025*	*0.007*
*osx*	*0.523*	0.375	−0.391	−0.224		*0.002*	0.293	0.872	0.804	0.123
*ptch1*	−0.172	*−0.637*	0.161	−0.112	*0.659*		0.866	0.778	*0.005*	0.916
*col2a1a*	0.411	−0.110	0.308	0.024	−0.254	0.041		*0.009*	0.205	0.596
*csf1ra*	−0.041	0.143	0.058	0.194	0.040	0.069	*0.585*		0.310	0.395
*gli1*	0.053	*0.475*	0.374	*0.511*	−0.061	*0.612*	−0.305	0.246		*0.019*
*ptch2*	0.230	−0.365	*−0.476*	*−0.601*	−0.366	−0.026	−0.130	0.207	*0.531*	
								

**Young Mutant Bone**										
	*runx2b*	*col10a1*	*AP*	*TRAP*	*osx*	*ptch1*	*col2a1a*	*csf1ra*	*gli1*	*ptch2*

*runx2b*		*0.026*	0.176	*0.000*	0.420	0.169	0.425	0.205	0.754	0.244
*col10a1*	*−0.474*		0.177	0.664	*0.030*	0.448	*0.052*	0.785	0.734	0.132
*AP*	0.299	0.299		0.145	0.482	0.913	0.123	0.304	0.161	0.597
*TRAP*	*0.746*	0.098	−0.322		0.860	0.307	0.822	0.158	0.910	0.727
*osx*	0.181	*0.462*	−0.158	−0.040		*0.002*	0.083	0.928	*0.000*	0.845
*ptch1*	0.304	0.171	−0.025	−0.228	*0.622*		0.251	0.060	*0.000*	0.736
*col2a1a*	0.179	*0.419*	0.339	−0.051	−0.378	−0.256		*0.015*	0.553	0.521
*csf1ra*	−0.281	0.062	−0.229	0.312	−0.020	0.407	*0.512*		0.452	0.537
*gli1*	−0.071	−0.077	−0.309	−0.026	*−0.702*	*0.768*	0.134	−0.169		0.657
*ptch2*	−0.259	−0.332	0.119	0.079	−0.044	0.076	−0.144	0.139	0.100	
									

**Old WT Bone**										
	*runx2b*	*col10a1*	*AP*	*TRAP*	*osx*	*ptch1*	*col2a1a*	*csf1ra*	*gli1*	*ptch2*

*runx2b*		*0.028*	*0.051*	*0.008*	*0.001*	*0.000*	*0.013*	0.078	0.151	0.063
*col10a1*	*−0.549*		*0.007*	*0.001*	*0.000*	*0.005*	*0.000*	0.735	*0.000*	0.230
*AP*	*−0.496*	*−0.641*		0.175	*0.005*	0.229	*0.011*	0.182	*0.003*	0.630
*TRAP*	*−0.633*	*−0.735*	−0.357		*0.000*	*0.000*	*0.001*	0.195	*0.051*	*0.051*
*osx*	*0.752*	*0.832*	*0.664*	*0.904*		*0.002*	*0.000*	0.590	*0.004*	0.087
*ptch1*	*0.771*	*0.663*	0.319	*0.781*	*−0.708*		*0.000*	0.083	*0.049*	*0.037*
*col2a1a*	*0.606*	*0.867*	*0.618*	*0.768*	*−0.772*	*−0.775*		0.674	*0.005*	0.221
*csf1ra*	−0.453	0.092	0.351	−0.342	0.146	0.447	0.114		0.131	0.679
*gli1*	−0.376	*−0.873*	*−0.701*	*−0.496*	*0.681*	*0.499*	*0.664*	0.394		0.433
*ptch2*	−0.475	−0.318	−0.131	*−0.495*	0.441	*0.525*	0.324	−0.112	−0.211	
									

**Old Mutant Bone**										
	*runx2b*	*col10a1*	*AP*	*TRAP*	*osx*	*ptch1*	*col2a1a*	*csf1ra*	*gli1*	*ptch2*

*runx2b*		*0.025*	*0.011*	0.664	*0.000*	0.747	*0.000*	0.843	0.211	0.124
*col10a1*	*−0.699*		*0.000*	0.648	*0.025*	0.400	*0.005*	0.636	0.529	*0.006*
*AP*	*0.761*	*0.954*		0.498	*0.029*	0.740	*0.004*	0.586	0.298	*0.020*
*TRAP*	−0.157	−0.165	0.243		0.408	0.225	0.850	0.807	0.117	0.668
*osx*	*0.926*	*0.698*	*−0.685*	0.295		0.715	*0.000*	0.942	0.207	0.117
*ptch1*	0.117	0.300	−0.121	−0.422	−0.133		0.470	0.713	0.072	0.379
*col2a1a*	*0.967*	*0.807*	*−0.822*	0.069	*−0.915*	−0.259		0.674	0.343	*0.022*
*csf1ra*	−0.072	−0.172	0.197	0.089	0.027	−0.134	0.152		0.127	0.252
*gli1*	0.433	0.226	−0.366	0.528	−0.437	0.590	−0.336	0.516		0.824
*ptch2*	0.519	*0.795*	*−0.715*	−0.155	−0.528	−0.312	*−0.709*	0.400	−0.081	

Note.—Partial correlation coefficients (below diagonal) and *P* values (above diagonal) are shown for both genotypes at young (3–5 months) and old (10–15 months) stages. Values are italicized if significant at the ∼0.05-level.

Differences in correlated gene expression were substantially greater in older adults, with mutants exhibiting 17/45 significant pairwise correlations, compared with 31/45 significant correlations in age-matched WT animals (table 2). These data suggest a far more integrated expression network of bone markers in WT versus *crocc2* mutants, an assertion that was supported by the network analyses (fig. 6*C* and *D*). Four modules were recovered for older WT animals, which were characterized by a high degree of correlation both within and between modules. Alternatively, gene expression in older mutants was characterized by two distinct modules, consistent with a dissociated gene network. This idea was supported by the spatial localization of TRAP and AP activities in WT versus *crocc2* mutants. In WT animals the enzymatic signature of bone resorption (i.e., TRAP) and deposition (i.e., AP) was typically colocalized (fig. 6*C*, inset), as expected based on the literature (e.g., Albertson and Yelick 2007; Cooper et al. 2013), and the interconnected expression of these two factors in the network (e.g., linked by various bone markers, fig. 6*C*). Alternatively, TRAP and AP activities were conspicuously distinct in *crocc2* mutants (fig. 6*D*, inset), consistent with their dissociated expression in network-space (fig. 6*D*).

All in all, these genetic data complement the analysis of *crocc2* bone phenotypes (e.g., fig. 5), and suggest that dysmorphic bone shape in *crocc2* mutants is underlain by mis-regulated marker gene expression.

### *Crocc2* Is Required for Bone Plasticity

**Fig. 7.**
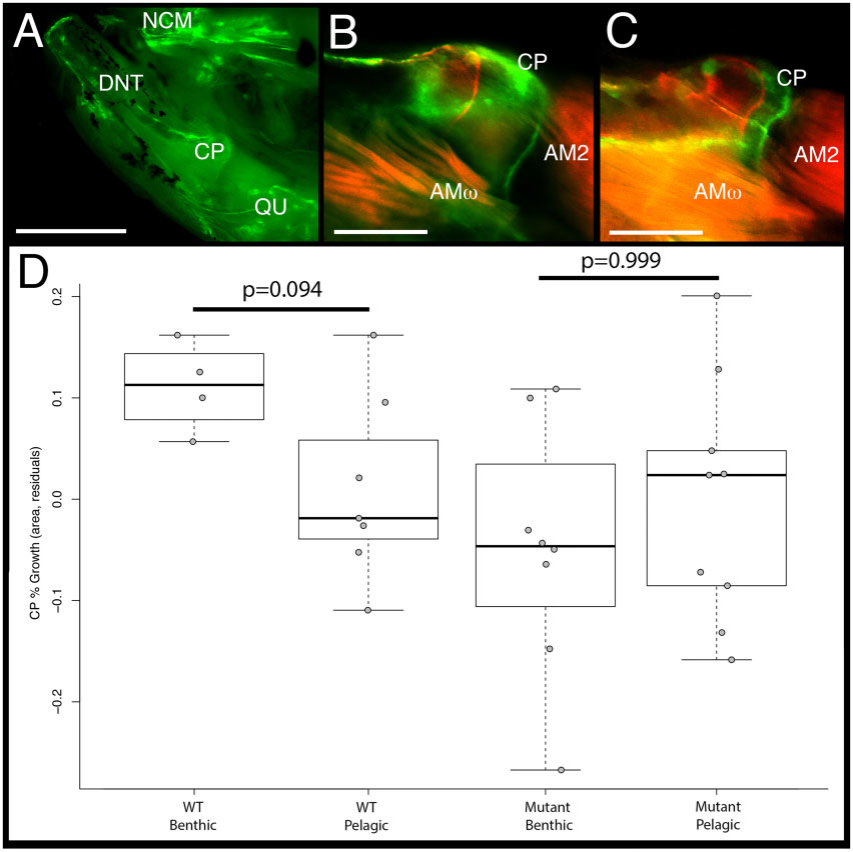
Rates of bone matrix deposition do not respond to environmental stimuli in *crocc2* mutants. Bone deposition rate was measured as the ratio between the area of the coronoid process (CP) at time 0 (red label) over the area at time 1 (green label) in WT and *crocc2* mutant zebrafish reared under alternate foraging regimes. Panel (*A*) shows the medial view of the oral jaw skeleton, under GFP illumination, depicting the anterior neurocranium (NCM), dentary (DNT), CP, and quadrate (QU). Scale bar for (*A*) equals 1 mm. Panel (*B*) depicts a composite image of red and green fluorochromes in the CP of a WT animal, whereas panel (*C*) shows the CP of a *crocc2* mutant. Two subdivisions of the adductor mandiblae can be seen in (*B* and *C*)—AM2 and AMω. Scale bars in (*B*) and (*C*) equal 200 µm. Panel (*D*) presents the results of a comparison of bone deposition rates. Pairwise significance was assessed via an ANOVA followed by a Tukey’s multiple comparison test.

To more explicitly test the hypothesis that *crocc2-*induced bone defects are due to impaired mechanosensing, we subjected fish to alternate feeding regimes intended to impose different functional demands on the craniofacial skeleton (fig. 7), similar to what was performed in cichlids (fig. 3). We then assessed rates of bone matrix deposition in the coronoid process (CP) of animals reared in different environments (fig. 7*B* and *C*). Our expectation was that WT animals would exhibit greater rates of CP bone deposition in the benthic foraging treatment where fish were required to leverage food from the substrate. We predicted further that this plastic response would be limited in c*rocc2* mutants. Our data supported both predictions: Rates of bone matrix deposition were higher in the CP from WT fish reared in the benthic versus pelagic treatment, and this response was absent in mutants (fig. 7*D*). Thus, mutant fish reared in the benthic environment appear to have lost the ability to deposit bone in response to increased mechanical load. More generally, these results are consistent with the hypothesis that dysmorphic bone phenotypes in *crocc2* mutants arise due to impaired mechanosensing.

During this analysis, we noted variation in CP shape, consistent with the results from the shape analysis described above. We therefore explored CP shape in these experimental animals, and found that it was distinct between treatments (Treatment: *Z* = 2.470, *P* = 0.003) and genotypes (Genotype: *Z* = 2.197, *P* = 0.005), and that there was a significant interaction effect between these two variables (Genotype-by-Treatment: *Z* = 2.194, *P* = 0.006). In addition, by quantifying shape using both fluorochrome labels, we were able to track shape over time, and document a significant effect of this variable on CP shape (Time: *Z* = 2.96, *P* = 0.001). Another notable outcome of this analysis was that WT shape, across time and treatments, exhibited relatively less variation compared with that across mutants, which occupied a far greater range of shape space (supplementary fig. S3, Supplementary Material online). This qualitative assessment was supported by quantitative tests of morphological disparity, which showed that mutants exhibited 2× the disparity as WT animals (0.0238 vs. 0.0119, respectively; *P* = 0.065). Increased disparity in mutant CP shape may be related to mis-regulated bone homeostasis (e.g., fig. 6).

## Conclusion

### Adaptive Radiations and the Root of Flexible Stems

Adaptive radiations constitute a major source of biodiversity on this planet, and have played a central role in our understanding of evolutionary processes. One attribute of adaptive radiations that has long intrigued and confounded biologists is their repeated, almost stereotypical, nature. For example, stem lineages that recurrently invade a novel environment (e.g., marine to freshwater among threespine stickleback) often diverge along highly predictable eco-morphological axes. Although similarities in ecological opportunity may explain some of these patterns, the extent to which replicate adaptive radiations are consistent has led to the proposition that other mechanisms may be at work. One notable hypothesis suggests that phenotypic plasticity in the stem lineage has the potential to bias the direction of adaptive radiations. Formalized as the flexible stem hypothesis (West-Eberhard 2003), this theory sets out to provide a mechanistic explanation for the repeated nature of adaptive radiations—for example, as an ancestral population is exposed to a novel environment, new phenotypic and genetic variants will be exposed to natural selection as individuals within the population mount a plastic response. Over time, those cryptic genetic variants that enable animals to more effectively exploit new resources may become fixed (i.e., genetic assimilation, sensu Waddington 1953), thereby biasing the direction of evolution along the eco-morphological axis established by the initial plastic response. Thus, if ancestral patterns of plasticity are similar across taxa, then the genetically fixed evolutionary responses should reflect that similarity. One empirical sign of such flexible stem evolution is predicted to be molecular similarity between morphological plasticity and evolution (Gibert 2017; Navon et al. 2020). Our work seeks to detect such signals.

We first set out to study cryptic genetic variation underlying cichlid jaw shape, with a focus on loci that underlie variation within distinct foraging environments. Fine-mapping implicated the *crocc2* locus, and functional studies in zebrafish supported the assertion that this gene is necessary for load-induced bone growth and remodeling. These results are consistent with the broader literature on the primary cilia and bone remodeling (Xiao et al. 2006; Papachroni et al. 2009; Qiu et al. 2012; Temiyasathit et al. 2012; Nguyen and Jacobs 2013). However, whereas the overwhelming majority of studies focus on the basal body, axoneme, and other more distal components of primary cilia, ours is unusual in implicating the proximal rootlets in bone biology. Whether the effect is due to ciliary integrity or a more nuanced, and as yet undescribed, role for the rootlets remains to be determined. Regardless of the specific mechanism, we showed that the African cichlid species with the divergent *crocc2* allele exhibited an assimilated phenotype—that is, high levels of bone matrix deposition regardless of mechanical environment. In the context of variation in the coiled-coil motif, this raises the interesting question of whether the number and/or integrity of the motif (i.e., fewer interruptions) might influence mechanosensing. In zebrafish, the loss (or reduction) of Crocc2 function resulted in reduced plasticity, supporting critical roles for this molecule in mechanosensing. In LF, loss of plasticity was associated with a putative gain-of-function polymorphism, where Crocc2 is characterized by fewer disruptions in the motif and correspondingly higher homodimerization affinity. Taken together, these insights suggest that the ability of bone cells to mechanosense may actually require a degree of interruption in the Crocc2 coiled-coil motif. In other words, this region of interrupted heptad repeats may serve to “sensitize” Crocc2/rootlets to environmental input. If true, this configuration may be actively selected for in African cichlids, several of which are known to be plastic in head/jaw shape (Parsons et al. 2014; Gunter et al. 2017; Hu and Albertson 2017; Navon et al. 2020).

This work constitutes the second in a set of experiments aimed at understanding the molecular basis of plasticity. The other has focused on Hh signaling (Parsons et al. 2016; Hu and Albertson 2017; Navon et al. 2020), which is notable given the close association between the primary cilium and the Hh signal transduction pathway. Members of the Hh pathway localize to the cilium (Yuan et al. 2015), and cells lacking cilia are unable to transduce a signal in response to the Hh ligand (Haycraft et al. 2005; Berbari et al. 2009). Thus, cilia have been said to constitute the “Hh signal transduction machine” (Goetz et al. 2009). Given the conservation of molecular mechanisms across vertebrates, understanding how, or if, Hh signaling and rootlets interact to effect bone biology in general, and mechanical load-induced plasticity in particular, could be a fruitful line of study. More generally, we suggest that the Hh-cilia signaling mechanism represents a robust molecular candidate for flexible stem evolution of the cichlid jaw.

## Materials and Methods

### Species and Husbandry

Both cichlids and zebrafish were used for this project. All cichlids were raised in 10 gal glass aquaria on standard flake food until 2 months of age, before being transferred to 40 gal glass aquaria. A single LF female was crossed to a single TRC male, creating a hybrid mapping population that was used for pedigree mapping. These species differ in craniofacial geometry and plasticity (Parsons et al. 2014; Albertson and Pauers 2019; Navon et al. 2020). A full-sibling F_1_ family was interbred to produce 25 F_2_ families, which were interbred to generate 265 F_3_ individuals used in this study. At 2 months, F_3_ families were split into two diet treatments, pelagic or benthic. For more detailed methods on these treatments and this cross, see previously published papers by Parsons and colleagues (Parsons et al. 2014, 2016). Briefly, a combination of flake food, algae wafers, and freeze-dried daphnia was ground and either sprinkled directly into the water column (pelagic treatment) or mixed with a ∼1–1.5% food-grade agar (Carolina Biological Supply Co., Burlington, NC) solution and spread over lava rocks (benthic treatment). Fish were raised to ∼7 months of age on each diet, euthanized with MS-222 according to approved IACUC protocols, fixed in 4% PFA, and stored in 75% ethanol. Prior to fixation, flank muscular tissue was taken for DNA extraction. Animals were dissected to reveal functionally salient bones and muscles, and imaged using a digital camera (Olympus E520).

Zebrafish were raised in 2.8-l plastic aquaria on a diet of rotifers from 5- to 12-day postfertilization, and then on a combination of GM-300 (Skretting) and brine shrimp thereafter. For the foraging experiment (details below), zebrafish in the pelagic treatment received GM-300 sprinkled directly into the water column, whereas benthic fish received GM-300 mixed with a ∼1% food-grade agar solution spread over the rough side of 2-in ceramic tiles. *Crocc2* mutant alleles were obtained from the Zebrafish International Resource Center (ZIRC). Allele 20707 consists of an ENU induced C > T nonsense mutation mapped to exon 8 that encodes a premature stop codon at amino acid 272. Allele 20708 contains a C > T nonsense mutation in exon 14 that creates a premature stop at amino acid 585. Fish harboring either allele yield comparable bone phenotypes; only 20707 phenotypes are reported here. Both alleles were contributed to ZIRC by the Stemple Lab (Busch-Nentwich et al. 2013) and map positions are based upon Zebrafish genome assembly GRCz11.

### Pedigree Mapping

QTL mapping methods and results are described elsewhere (Parsons et al. 2016; Zogbaum et al. 2021). Briefly, genomic DNA was extracted from flank muscle tissue using DNeasy blood and tissue kits (Qiagen Inc., CA), digested with the SbfI restriction enzyme, processed into RAD libraries as described (Chutimanitsakun et al. 2011), barcoded and sequenced using an Illumina HiSeq 2000 (Illumina, San Diego, CA) and single-read (1 × 100 bp) chemistry. We focused on a locus for lower jaw mechanical advantage, which mapped to an interval on linkage group (LG) 21, with a peak genotype–phenotype association at a marker on physical scaffold 31 at 2,946,476 bp (fig. 2*A*). Since an F_3_ hybrid cross allows for a relatively higher number of recombination events and mapping resolution, we used additional, unmapped, RAD-seq SNPs to assess genotypic effects along this scaffold at increasingly fine scales, using makers every ∼0.5 Mb (fig. 2*C*) and ∼0.1–0.2 Mb (fig. 2*D*). In addition, genetic divergence between wild-caught LF and TRC (imported directly from the lake) was explored, using a panel of 3,087 RAD-seq SNPs, and *F*_ST_ values following Nei (1987) and calculated in the R package HIERFSTAT. These fishes were genotyped following the same RAD procedures and SNP calling pipeline, and at the same time as the hybrids.

### Immunohistochemistry

Immunostaining was performed with mouse anti-acetylated alpha tubulin (1:500; Sigma T6793) or rabbit anti-gamma tubulin (1:500; Sigma T6557). Amplification of T6793 signal was performed using donkey anti-mouse Biotin (1:100) and Alexa 488 Streptavidin Conjugate (1:1,000) (Jackson Immunoresearch). Donkey anti-rabbit Alexa 594 was used to visualize gamma tubulin antibodies. Briefly, animals were anesthetized and sacrificed using MS222 (Western Chemical, Inc.) and fixed for 1.5 h in 4% paraformaldehyde, pH 7.4, at room temperature. For young zebrafish, 4dpf larval samples were permeabilized in acetone at −20 °C for 20 min followed by 1% Triton X-100 in PBS for 1 h, and blocked in 5% donkey serum (Jackson Immunoresearch) in 0.1% Triton X-100 in PBS. For adult zebrafish, samples were embedded in 1.5% agar/5% sucrose and 20 μm cryosections were blocked for 1 h before immunostaining. All Washes were performed in 0.1% PBS-Tween 20, pH 7.4. To prevent photobleaching, all samples were mounted using Vectashield with DAPI (H-1200; Vector Labs).

### Geometric Morphometrics

Adult zebrafish were cleared and stained using traditional methods (Potthoff 1984; Taylor and Van Dyke 1985). All dissections, and subsequent imaging, were performed using a Leica M165 FC microscope, and attached Leica DFC450 camera (Leica Camera AG, Wetzlar, Germany). We imaged the lateral profile of the lower jaw, and dorsal surface (when premaxillae are protruding) of the kinethmoid (Hernandez et al. 2007) for morphological analyses. Geometric morphometric data were collected using Stereomorph (Olsen and Westneat 2015) in R (R Core Team 2018). In total, we summarized the lower jaw using six fixed and four semilandmarks (sliding) and the kinethmoid using four fixed and eight semilandmarks (see Rohlf and Slice 1990; Gunz and Mitteroecker 2013, for more information on fixed/semilandmarks).

Morphological data were aligned via generalized Procrustes superimposition (Goodall 1991) and then analyzed via ANOVA to test for significance differences in mean shape between homozygous genotypes for both the lower jaw and kinethmoid. In all analyses, we compared null models (shape ∼ size) to full models (shape ∼ size + genotype) to control for the effects of size. Tests were conducted utilizing a randomized residual permutation procedure (RRPP) and the data were subjected to 10,000 random permutations (Collyer and Adams 2018; Collyer et al. 2015). All morphological analyses were performed using Geomorph v3.1 (Adams et al. 2015, 2018).

### Quantitative Real-Time PCR and Network Analysis

We purified RNA from homogenized whole heads of zebrafish excluding eyes and brain, between the ages of 3 and 15 months, in Trizol (Invitrogen) using phenol–chloroform. We standardized resulting cDNA to 70 ng/μl using a High-Capacity cDNA Reverse Transcription Kit (Applied Biosystems). To determine relative gene expression levels, we used a 10-μl total reaction in triplicate using a QuantStudio3 Real-Time PCR System (Applied Biosystems). Each gene assessed was compared with expression levels of β-actin to determine relative expression levels via the ΔΔCT method (Livak and Schmittgen 2001). Sample size was *n* = 5 for all genes in each age group/genotype except 10- to 15-month (i.e., old) mutant *ptch2* where *n* = 4. We used ANOVA for statistical analyses in R.

In order to determine the covariation of gene sets in our quantitative real-time PCR (qPCR) data set, we constructed gene networks in R. First, we used pairwise partial correlations with the ppcor package using the Pearson method to account for multicollinearity (table 2). We next used the iGraph package to perform and visualize network analyses for each data set. These analyses weight the relationships between each gene based on the pairwise partial correlation value strengths. Correlations with a *P* value below 0.15 were included in the construction of the gene networks (fig. 6). The number of lines between each pair of genes indicates the strength of the covariation between them (i.e., five lines represents stronger correlation than 2).

### Bone Deposition Analysis

Bone deposition experiments are described in detail elsewhere (Navon et al. 2020). Briefly, fish were anesthetized using MS-222 in cool water during injections and handling. They were injected with alizarin red (50 mg-fluorochrome/kg fish) at the first timepoint and with calcein green (0.5 mg-fluorochrome/kg fish) at the second timepoint, approximately 5 weeks apart. One week after the final fluorochrome injection, fish were euthanized with a lethal dose of MS-222 and stored in 95% ethanol at 4 °C. Craniofacial bones and flank scales were dissected from the head and body, cleaned of surrounding soft tissue, and flat mounted on glass slides. Cichlid bones were imaged with a Zeiss Axioplan2 fluorescent apotome microscope. Zebrafish bones were imaged with a Leica M165 FC microscope, and attached Leica DFC450 camera. All elements were imaged in triplicate using a red fluorescent filter, a green fluorescent filter, and a DCIM bright-field view. Trunk scales were flat mounted and imaged in the same way. Bone deposition was quantified by calculating the distance between the red and the green fluorochrome labels in each bone using Photoshop. Bone deposition was standardized for individual growth rate by regressing bone growth on scale growth and taking the residual values for downstream analysis. A series of ANOVAs using treatment and species (cichlids) or genotype (zebrafish) were performed in R (R Core Team 2018). Tukey’s post hoc analyses (i.e., TukeyHSD) were performed to identify significant pairwise differences.

## Supplementary Material

Supplementary data are available at *Molecular Biology and Evolution* online.

## Supplementary Material

msab071_Supplementary_Data
