# Supplementary material for: Ciliary Rootlet Coiled-Coil 2 (crocc2) Is Associated with Evolutionary Divergence and Plasticity of Cichlid Jaw Shape
Source: Mol Biol Evol. 2021 Mar 15;38(8):3078–92. doi: 10.1093/molbev/msab071 (PMC8321518; doi:10.1093/molbev/msab071)
Supplement: msab071_Supplementary_Data [file msab071_supplementary_data.zip › crocc2 MS SI.pdf]

Danio rerio qRT-PCR Primer Sets	
**Gene Name**	**Sequence (5'-3)'**
Runx2b For	CAAACACCCAGACCCTCACT
Runx2b Rev	GTATGACCATGGTGGGGAAG
Osx For	GCGTCGATTCTGGAGGAG
Osx Rev	AATCTCGGACTGGACTGGTG
AP For	CAGTGGGAATCGTCACAACAA
AP Rev	CAACACAGTGGGCATAAGCA
Csf1ra For	GGCCAGCATAAGAACATCGT
Csf1ra Rev	CGTCATGGGTTCTGGAAAGT
TRAP For	CGTCCACTGACCACAGGAAGA
TRAP Rev	AAGGATCCTGACGTCTGATTGA
Col10a1 For	CCTGTCTGGCTCATACCACA
Col10a1 Rev	AAGGCCACCAGGAGAAGAAG
Col2a1a For	ATCCCATCATTTCACCTGGA
Col2a1a Rev	TCTGTCCCTTTGCACCAAGT
Ptch1 For	GCCGCATCCCAGGCCAACAT
Ptch1 Rev	CGTCTCGCGAAGCCCGTTGA
Ptch2 For	CATCCCATTCAAGGAGAGGA
Ptch2 Rev	GGCAGGGAATATCAGCAAAA
Gli1 For	GTCATCCGCACCTCTCCAAA
Gli1 Rev	ATGGTGCCCACAGACAGATG
B-Actin For	CAACAGGGAAAAGATGACACAGAT
B-Actin Rev	CAGCCTGGATGGCAACGT

Table S1. Primer sequences for zebrafish bone markers and the house-keeping gene, b-actin.

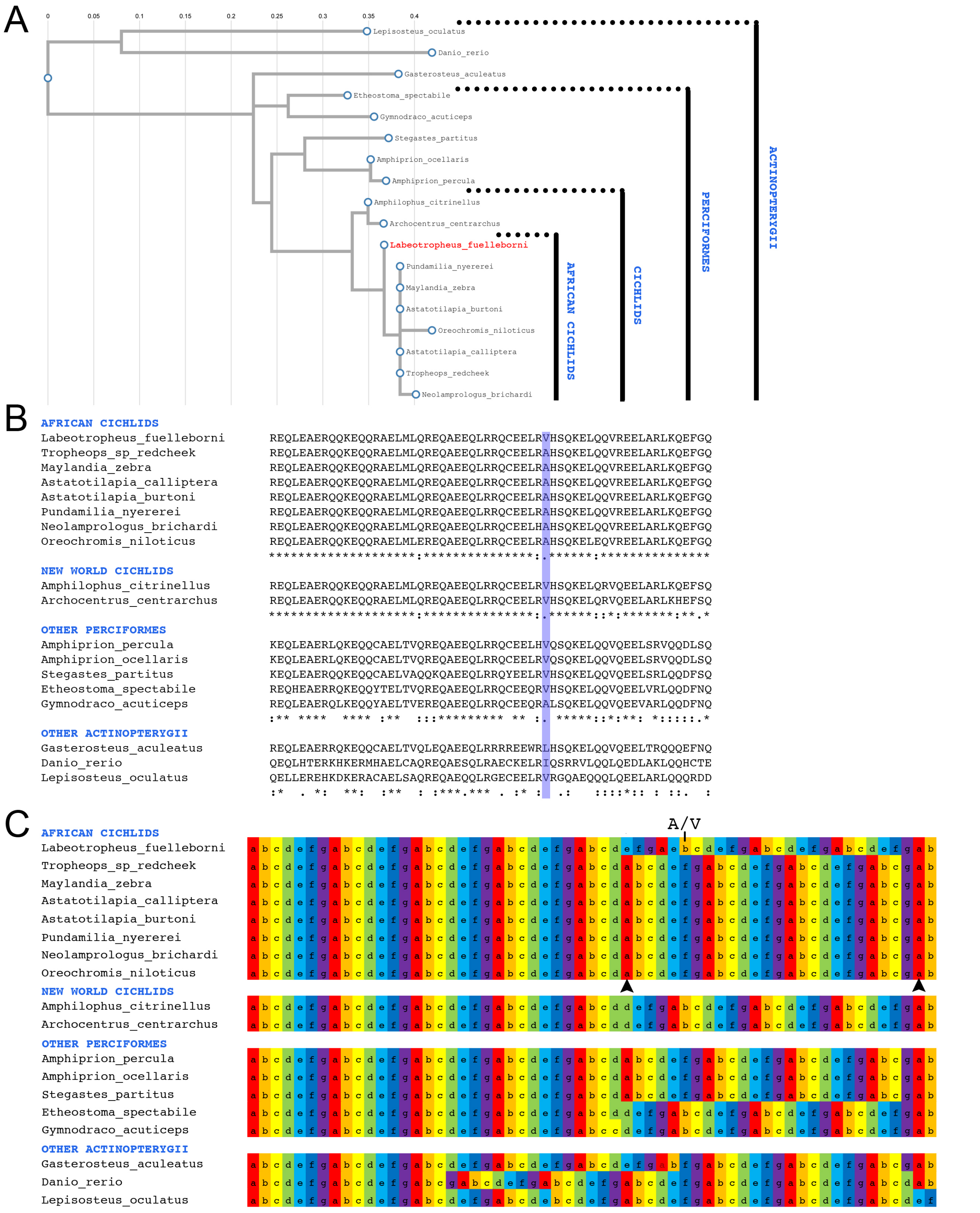

Figure S1. Sequence variation in Crocc2 across fishes. Amino acid sequence around the coding mutation identified in our genetic cross is highly conserved across African cichlids, but sequence homology begins to breakdown when other perciform and ray-finned fish species are included in the alignment (A-B). Notably, whereas all other

African cichlids examined have an Alanine (A) at position 963, *Labeotropheus* has a Valine (V), which is similar to New World cichlids as well as most other perciform species. In fact, all perciforms possess either an A or V, and all fish species possess a non-polar, hydrophobic amino acid at this position (blue shade, B). While considerable sequence variation exists across this stretch of amino acids, the resulting coiled-coil motif is largely conserved, especially toward the N-terminus (C). The V in *Labeotropheus* results in few interruptions (black arrowheads) in heptad repeats (i.e., a-g) compared to other African cichlids (C). This region of Crocc2 is also associated with greater predicted structural variation, which suggests that this portion of the protein may be less constrained, and/or a target of natural selection. All Crocc2 sequences were obtained from NCBI and the Ensembl genome browser.

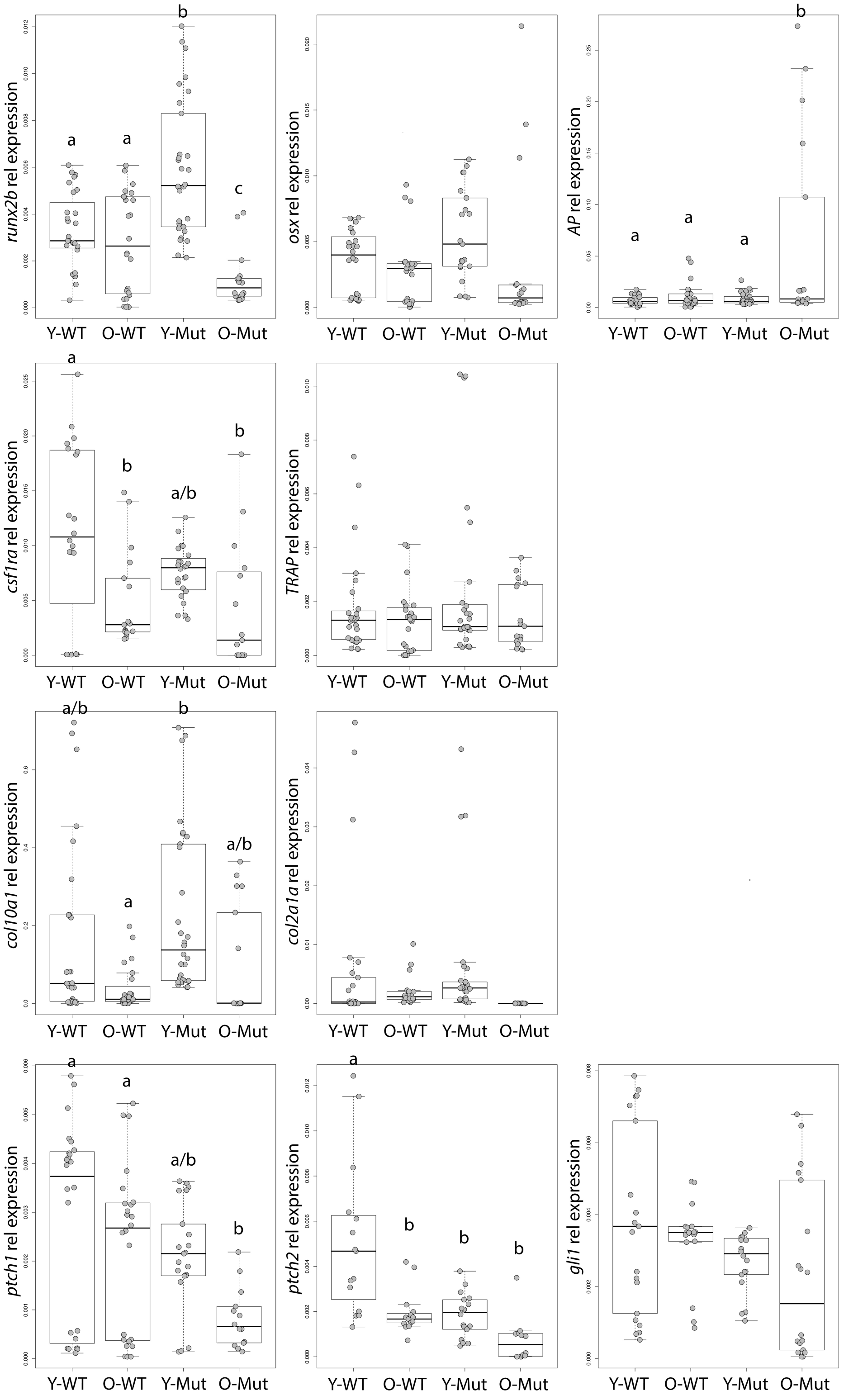

Figure S2. Expression results from bone marker genes. Full results are shown for quantitative RT-PCR, organized in the same pattern as Table 1. Relative expression levels are shown for young adult WT (Y-WT), old adult WT (O-WT), young adult *crocc2* mutants (Y-Mut), and old adult *crocc2* mutants (O-Mut). Letters above the box plots refer to statistical groupings as determined by ANOVA followed by a Tukey's multiple comparison test. Graphs with no letters, did not exhibit any significant pair-wise differences.

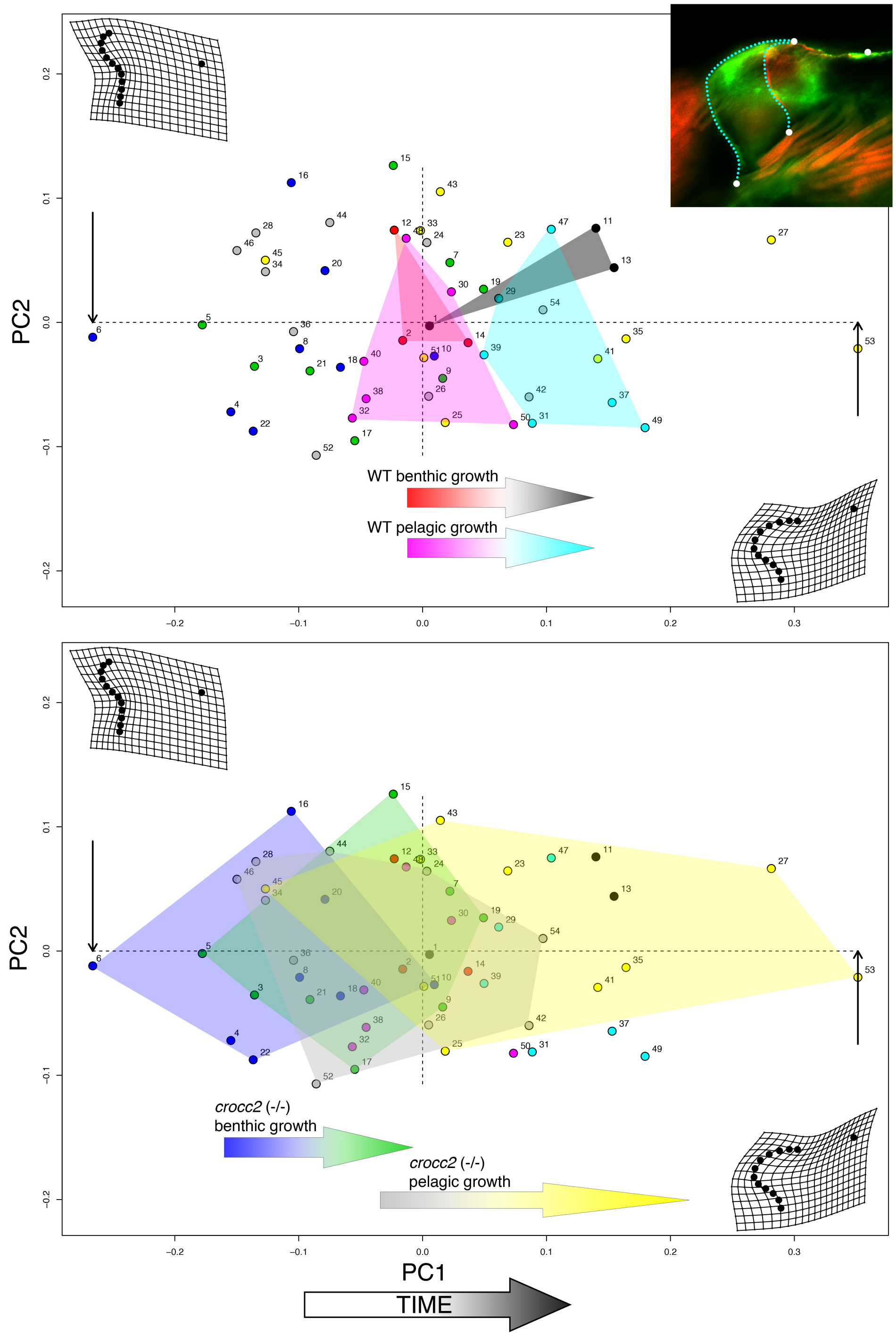

Figure S3. Shape analysis of the CP in *crocc2* and WT zebrafish across environments. Shape space from a morphometric analysis on CP shape. PC1 accounts for 63% of the variation, and mainly captures variation in growth over time, such that shape at time 0 (determined using the red fluorochrome, Alizarin Red) is associated with more negative PC1 scores, whereas shape at time 1 (green fluorochrome, Calcein Green) is associated with more positive PC1 scores. PC2 accounts for 17% of the variation in CP shape. The inset at top illustrates the digitizing scheme, with landmarks depicted as white dots and semi-landmarks arrayed along the blue dotted line. Note that each animal is measured twice - once for T0 shape (red), and once for T1 shape (green). In shape space, T1 and T0 are numbered consecutively, such that samples 1 and 2 correspond to individual one at times 1 and 0, respectively.
